# Simplified bionic solutions: a simple bio-inspired vehicle collision detection system

**DOI:** 10.1088/1748-3190/aa5993

**Published:** 2017-02-15

**Authors:** Manfred Hartbauer

**Affiliations:** Institute of Zoology, University of Graz, Universitätsplatz 2, 8010, Austria

**Keywords:** collision detection, image processing, obstacle avoidance, DCMD neuron, locust, driver assistant

## Abstract

Modern cars are equipped with both active and passive sensor systems that can detect potential collisions. In contrast, locusts avoid collisions solely by responding to certain visual cues that are associated with object looming. In neurophysiological experiments, I investigated the possibility that the ‘collision-detector neurons’ of locusts respond to impending collisions in films recorded with dashboard cameras of fast driving cars. In a complementary modelling approach, I developed a simple algorithm to reproduce the neuronal response that was recorded during object approach.

Instead of applying elaborate algorithms that factored in object recognition and optic flow discrimination, I tested the hypothesis that motion detection restricted to a ‘danger zone’, in which frontal collisions on the motorways are most likely, is sufficient to estimate the risk of a collision. Furthermore, I investigated whether local motion vectors, obtained from the differential excitation of simulated direction-selective networks, could be used to predict evasive steering maneuvers and prevent undesired responses to motion artifacts.

The results of the study demonstrate that the risk of impending collisions in real traffic scenes is mirrored in the excitation of the collision-detecting neuron (DCMD) of locusts. The modelling approach was able to reproduce this neuronal response even when the vehicle was driving at high speeds and image resolution was low (about 200 × 100 pixels). Furthermore, evasive maneuvers that involved changing the steering direction and steering force could be planned by comparing the differences in the overall excitation levels of the simulated right and left direction-selective networks. Additionally, it was possible to suppress undesired responses of the algorithm to translatory movements, camera shake and ground shadows by evaluating local motion vectors. These estimated collision risk values and evasive steering vectors could be used as input for a driving assistant, converting the first into braking force and the latter into steering responses to avoid collisions. Since many processing steps were computed on the level of pixels and involved elements of direction-selective networks, this algorithm can be implemented in hardware so that parallel computations enhance the processing speed significantly.

## Introduction

The European ‘White Paper on Road Safety’ proposed several measures to improve vehicular safety and stressed active safety and automated driving measures. Active safety systems take control of a vehicle in hazardous situations and, if effective, are significantly beneficial in that they prevent fatalities. The most effective system for the prevention of fatal accidents consists of automated collision avoidance with emergency steering and braking, as well as active lane keeping assist systems (e.g. Vahidi and Eskandarian (2003) and Eichberger (2011)). Driving automation requires fast, reliable systems that are able to make quick decisions in the case of an emergency, where reaction time is critical. This requires sophisticated sensor systems, robust signal processing and algorithms that facilitate quick decision-making for vehicle control. One of the main weaknesses of current collision avoidance systems is that they depend on various expensive sensors and camera systems that have high computational power demands. For example, the driverless cars developed by Google™ use an expensive 64-beam laser scanner to make 3D scans of the environment. Presenting a possible alternative to the existing collision-avoidance systems that are based on complex radar technologies and laser scanning techniques, this study describes a rather simple, collision detection (CD) algorithm that was inspired from the visual information processing of locusts. Locusts are well known for the efficient collision avoidance behavior they display on the basis of visual cues when flying in large swarms (Santer *et al* 2005, 2006, 2012, Rind *et al* 2008, Chan and Gabbiani 2013) or when stationary (Santer *et al* 2005, 2007, Fotowat and Gabbiani 2007). In the neurophysiological part of this study, the spiking activity of a collision-detector neuron was recorded during the presentation of several dangerous traffic scenes. Since the collision risk was reliably encoded in the nervous activity, I aimed to simulate the activity of this neuron by means of a novel CD algorithm that evaluates the motion occurring in a zone in which frontal collisions are most likely. Although this simple CD algorithm is not based on the function of neuronal networks, it was able to reproduce the activity of a collision-detector neuron, a result that may inspire the development of novel collision avoidance systems.

One challenge associated with the development of collision avoidance systems based on a single camera is the fact that the visual surroundings are represented in only two dimensions as opposed to stereo cameras enabling the computation of disparity maps (Pasquale *et al* 2016). This makes it difficult to discriminate objects appearing on a collision course on the sole basis of camera frame sequences that include pass-by objects (e.g. on-coming traffic in the opposing lane) and the optic flow. Therefore, it is considered essential to divide the environment into regions of coherently moving objects to enable intelligent, autonomous navigation, especially in the cases of landing maneuvers and collision avoidance (Zufferey 2009). To accomplish this challenging task, the optical flow constraint equation and depth-velocity ambiguity can be solved by means of complex mathematical algorithms (Kolodko and Vlacic 2005). Alternatively, when relying on a single camera system, certain object features such as edges that radially expand and the contrast change that occurs during object looming can be examined to determine whether objects are on a collision course (Rind and Simmons 1992, Judge and Rind 1997, Chan and Gabbiani 2013). Gregarious locusts with limited stereovision use this type of collision detection to avoid impending collisions (Blanchard *et al* 1999, 2000, Shigang and Rind 2005, Yue and Rind 2006a, 2006b, 2007).

**Figure 1:**
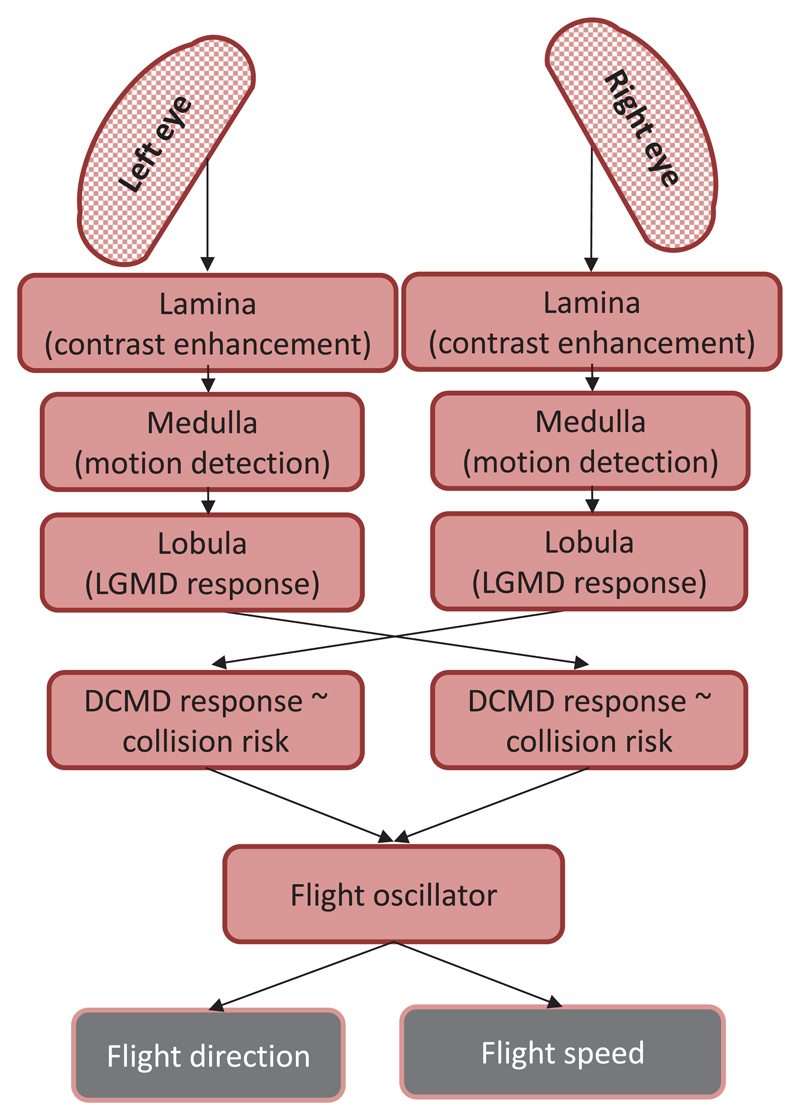
Illustration of visual information processing in the locust. LGMD and DCMD response is related to the collision risk. Note that the activity of the LGMD neuron is mirrored in the activity of the DCMD neuron of the contralateral side.

**Figure 2:**
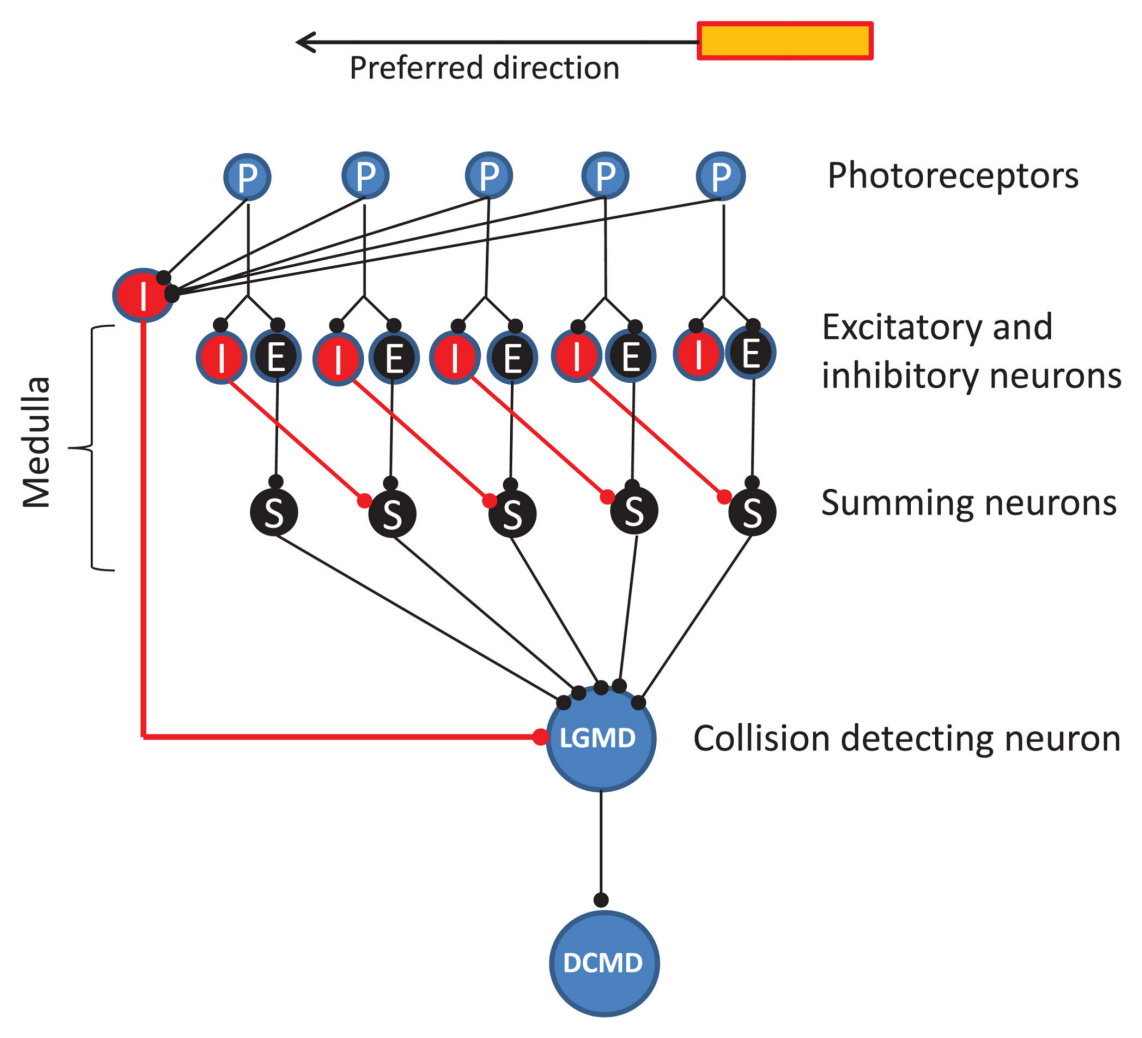
Illustration of the working principle of elementary motion detectors (EMDs). A moving light bar subsequently stimulates neighboring photoreceptors of the compound eye. Receptor excitation is indirectly conveyed to EMDs that extract the information about object motion (direction and velocity). Excitatory neurons and inhibitory neurons are connected with summing neurons. Since lateral inhibition is delayed relative to excitation, a response of summing neurons is restricted to the left-ward motion of the light bar (preferred direction). On the contrary, delayed lateral inhibition suppresses the response of summing neurons when the bar moves from left to right. Subsets of EMDs with different ‘preferred directions’ synapse with the LGMD neuron that is connected with the DCMD neuron. The activity of the LGMD neuron is suppressed upon simultaneous activation of all receptors (inhibitory neuron on the left side).

The eyes of the locust species *Locusta migratoria* and *Schistocerca gregaria* are composed of hundreds of tightly packed ommatidia that form an apposition compound eye that sense the environment at a low level of spatial resolution. Important steps of visual information processing in the optic lobe of locusts are illustrated in figure 1. A first important step of visual information processing takes place in the lamina, where the edge contrast of the visual stimulus is enhanced by means of a center/surround inhibition (Fotowat and Gabbiani 2007). Motion information is further processed in the medulla, where the interaction between local excitation and delayed lateral inhibition from parts of the peripheral receptive field leads to the directional sensitivity of elementary motion detectors (EMDs) (Rind and Bramwell 1996; see illustration in figure 2). Information from the EMDs is fed forward to a wide-field neuron called the lobula giant movement detector (LGMD), which forms a synapse with the descending contralateral movement detector (DCMD, or so-called ‘collision-detector neuron’) neuron (Rind 1984, Rind and Simmons 1992, Rind and Bramwell 1996). The DCMD neurons only receive visual information from one eye and selectively respond to incoming/expanding objects that are on a collision course (Judge and Rind 1997, Rind and Simmons 1997, Guest and Gray 2006, Gray *et al* 2010). When the firing rate of this neuron exceeds 150 Hz, gliding is triggered in flying locusts (Santer *et al* 2005, 2006, Rind *et al* 2008).

The ways locusts and flies extract visual cues related to impending collisions inspired the development of collision detectors using several bionic approaches. For example, mobile robots have been equipped with silicon eyes to demonstrate that locust-like visual motion processing is sufficient for collision avoidance (Shigang and Rind 2005, Yue *et al* 2009, Yue and Rind 2012, Pericet-Camara *et al* 2015). Furthermore, motion-sensitive cameras have been developed by simulating the neuronal network of insects engaged in visual motion processing (Moini *et al* 1997, Okuno and Yagi 2007). Discrimination between translating objects that display highly directional motion from colliding objects with radially expanding motion constitutes a challenging task and can be achieved by simulating whole-field direction-selective neuronal networks (Yue and Rind 2006b, 2013a, 2013b). However, realistic simulations of neuronal networks are computationally demanding and involve extensive parameter tuning, which can be achieved by using evolutionary optimization algorithms. Therefore, the question arises whether a simple numeric simulation of the function of neuronal networks may yield similar results.

In this study, movies of traffic scenes that were recorded with dashboard cameras mounted in cars were shown to locusts in order to study the response of the DCMD neuron to impending collisions and everyday traffic situations. Surprisingly, this neuron selectively responded to impending collisions, but rarely to normal (i.e. harmless) traffic situations. Therefore, I developed a rather simple numeric algorithm to reproduce the DCMD response in a novel modelling approach that deviates from classic approaches which are usually based on the optic flow field (Bruhn *et al* 2005, Liu *et al* 2011, Song *et al* 2011, Franceschini 2004), discrimination of foreground against background (Kim *et al* 2005, Spagnolo *et al* 2006) and object recognition (Kolodko and Vlacic 2005). This bionic CD algorithm is not based on what is known about the neuronal processing of visual information in locusts, instead it aims to estimate the risk of impending collisions simply by evaluating the motion in an area in which frontal collisions are most likely to occur (i.e. the ‘danger zone’). Results showed that this bionic algorithm could be used to estimate the risk of impending collisions although only low-resolution movies (about 200 × 100 pixels) of fast driving cars were used as input. Furthermore, I simulated the function of the lateral inhibitory networks in a simple way to extract local motion information for the calculation of plausible evasive steering maneuvers and suppress undesired responses to camera shake and ground shadows.

## Methods

### Traffic films

Traffic films that were used in this study for neurophysiological experiments and the development of a novel collision detection (CD) algorithm were recorded using dashboard- or windscreen-mounted cameras in cars that were rapidly driven in a variety of traffic situations. Video footage was downloaded from car crash compilations from the Youtube™ platform. Original color movies were converted into Windows Media Video (.wmv) format using the Youtube Movie Maker™. Eight different traffic films were presented continuously (in a loop mode) to locusts, such that the visual information was received only by the right eye, in the neurophysiological experiment. Each scene was repeated 11–20 times while the activity of the left DCMD neuron was recorded using a hook electrode positioned between the head and thorax. Traffic films were presented at a frame rate of 30 Hz via a tablet PC (Samsung Galaxy Tab2, 10.1) that was positioned 10 cm from the locust.

All movies that were broadcast to the locusts were also used as input for the development of a novel collision-detection (CD) algorithm. For this purpose, traffic films were converted into a sequence of grey-scale movie frames using VirtualDub (Version 1.9.11). In order to match the low resolution of insect compound eyes, the spatial resolution of the movie frames was reduced to about 200 × 100 pixels (depending on the aspect ratio). All the traffic films had a frame rate of 29 frames s^−1^ (fps), except the crash involving a pedestrian where frame rate was only 14 fps.

### Neurophysiology

Individuals of *Schistocerca gregaria* were anaesthetized using chlorethylene and mounted ventral-side up on a metal insect holder. All legs were removed and both neck connectives were exposed with the help of fine surgical instruments. A steel reference electrode was inserted into the abdomen and a tungsten-wire hook electrode was used to lift the left connective and record the neuronal activity. After removing the hemolymph, desiccation of the connective was prevented by sealing the wound with petroleum jelly. All playback experiments were performed in a dark chamber with the following dimensions: 90 cm × 80 cm × 80 cm. A customized bio-signal amplifier, modified from an amplifier described by Land *et al* (2001), was used to amplify electrode signals against the reference electrode. Analog to digital conversion was performed using a Powerlab/4s converter (AD Instruments, Spechbach, Germany), operated at a sampling rate of 10 kHz. The software Chart (Version 5, AD Instruments, Spechbach, Germany) was used to record the electrode signals, and brief sound signals were used to mark the beginning of a film sequence. In this way, it was possible to time the spiking response and compare this with the traffic film sequence. The action potentials of the DCMD neuron are characterized by their high extracellular discharge amplitude, which allowed me to discriminate the activity of this unit from that of other units by defining an amplitude threshold. Spike sorting and the calculations of the average spike rate were performed using Spike2 (v5.2.1, Cambride Electronic Design, UK). The mean spike rate frequency obtained during film presentation was calculated in a gliding time window with a 0.3 s duration. Since the aim of this study was to develop a novel CD algorithm, films were only presented to three individuals and the DCMD response was evaluated qualitatively, rather than quantitatively. A luxmeter (Ahlborn Inc. Swiss, model: SL A613-VL, Serial number: 4090042) was used to measure the average brightness of the films at the locust’s position (results are summarized in table 1).

### The Netlogo approach

In previous simulation studies (e.g. Stafford *et al* (2007), Shigang and Rind (2005), Yue *et al* (2009), Yue and Rind (2012) and Yue and Rind (2013b)), neurons of simulated networks exhibited rather realistic properties with respect to the neuronal architecture, membrane potential dynamics and synaptic weights. Instead of simulating all these details, only the functional role of the photoreceptors and neuronal networks were modelled in a numeric model using the open-access multi-agent modelling software Netlogo (Version 5.3.1; http://ccl.northwestern.edu/netlogo/). This software allows creating elements (also called ‘turtles’) to act as ‘photoreceptors’ or ‘neurons’ in a lateral inhibitory network. The main advantage of this modelling environment is that the algorithm and simulation parameters can be easily changed and efficiently tested. The parallel processing capabilities of this software facilitates the embedding of the CD algorithm in future hardware solutions, such as field programmable gate arrays (FPGA) chips (e.g. Aubépart and Franceschini (2007)). Once the CD model demonstrated to indicate the risk of an impending collision and predict plausible collision avoidance vectors, the simulation parameters were optimized with the help of the ‘behavior space’ function available in Netlogo. Using this function, it is possible to systematically change a set of parameters and save the simulation result (collision risk) after processing a certain number of frames. The algorithm described in this study uses a sequence of low-resolution images as input and all simulation parameters were optimized to extract undirected and directed motion in a reliable manner. For control purposes, films showing every-day traffic situations such as on-coming traffic, a ‘crash-free’ high speed drive on a highway and overtake maneuvers were used as input for the CD algorithm. The simulation parameters of the CD algorithm were optimized to indicate impending collisions reliably while showing little or no response to everyday traffic situations. Since the contrast of these films varied greatly, it was necessary to adjust the threshold of the minimal luminance change contributing to the estimated collision risk for each film (see table 1). All other simulation parameters, except the size and position of the ‘danger zone’, were left unchanged irrespective of which film was used as an input. Since Netlogo restricts the range of grey values to floating point numbers in the range between 0 and 9.9, the thresholds and derived receptor potentials (see below) refer to the grey values within this range.

### Overview of methods

**Figure 3:**
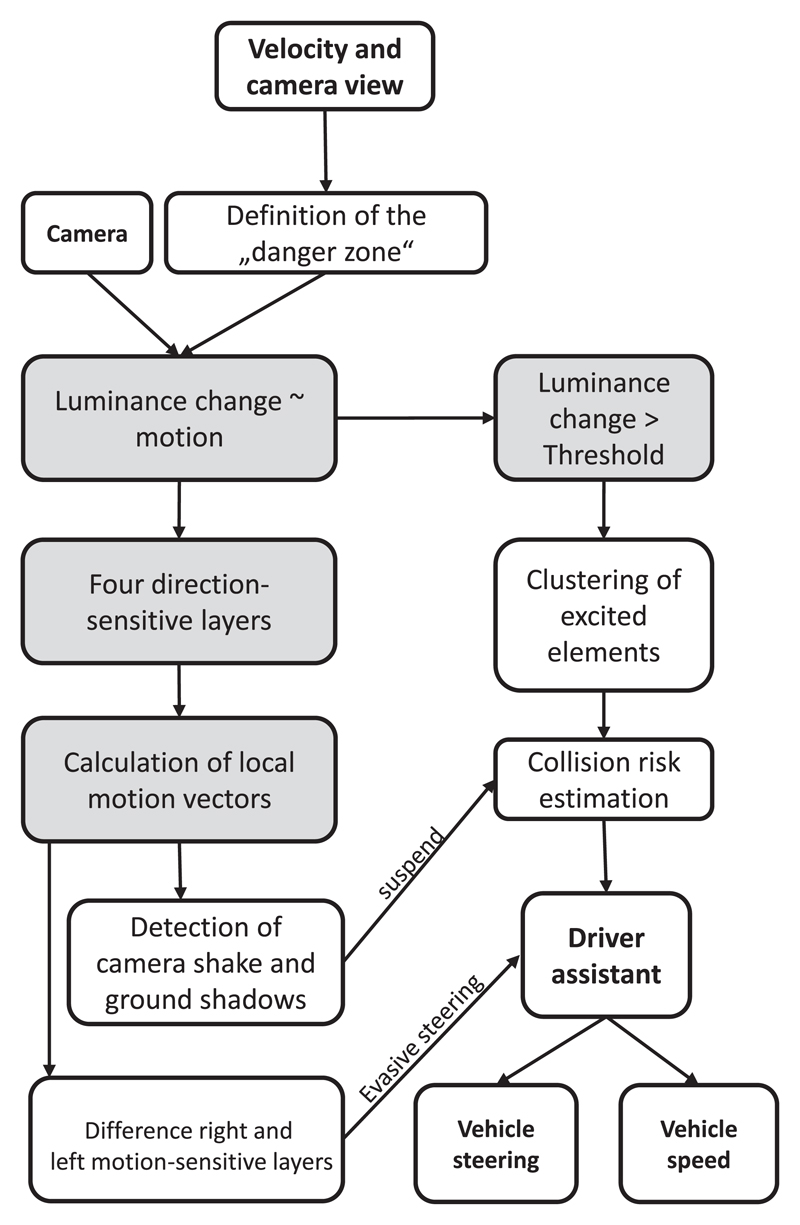
Method overview. Outline of the CD algorithm and method used to calculate evasive steering directions and undesired responses to ground shadows. The grey background indicates parts of the algorithm that were computed at the level of elements (simulated receptors and neurons).

An overview of the processing steps that were used to calculate the collision risk, plan evasive steering maneuvers and suppress responses to camera shake and ground shadows is illustrated in figure 3. The speed of the cars and the view of the dashboard camera were used to define a ‘danger zone’, within which the collision risk was calculated on the basis of luminance changes. All excited receptor elements needed to be clustered in order to prevent undesired responses of the CD algorithm to small or lengthy objects. Excited receptors stimulated elements of direction selective layers, each of which responded preferably to object motion that was directed upward, downward, left or right. This sensitivity to the direction of motion was the consequence of delayed lateral inhibition, a mechanism that shapes the direction-sensitivity of EMDs of locusts (see figure 2; Rind and Bramwell 1996). By comparing the responses of only four motion-selective layers, it was possible to estimate the local motion vectors that could be used to compute possible evasive steering vectors, suppress responses to camera shake and ground shadows (see below).

### Estimation of the ‘danger zone’

Calculations of the estimated collision risk and the evasive steering vectors were restricted to an image region in which it would be necessary to quickly respond to approaching objects to avoid collisions with frontally approaching objects (i.e. the ‘danger zone’). The dimensions of this circular image area depend on the speed of the vehicle, and its position depends on the driving direction relative to the camera perspective. The lower the speed of the vehicle, the larger the dimensions of this zone and vice versa. In principal it would be possible to calculate the ‘danger zone’ on the basis of optic flow calculations (Bruhn *et al* 2005, Liu *et al* 2011, Song *et al* 2011) by restricting this zone to an area in which optic flow remains rather low. In this study, however, the size and position of the danger zone was manually set to estimate the collision risk and left unchanged while processing the film (see table 1 for dimensions). This method is based on the assumption that driving speed and steering is permanently available in modern cars and the camera perspective, image size and aspect ratio of a firmly mounted windscreen camera remains constant. Therefore, the ‘danger zone’ can be calculated real-time after performing a simple calibration procedure. To suppress possible responses of the CD algorithm to rapidly approaching ground shadows and lane markings, the lower region of the movie (1/4 of the frame height) was excluded from the ‘danger zone’. Furthermore, to exclude responses to bridges and overhead signs, the upper frame region (1/4 of the frame height) was also excluded from the estimation of collision risk.

### Quantification of object motion

**Figure 4:**
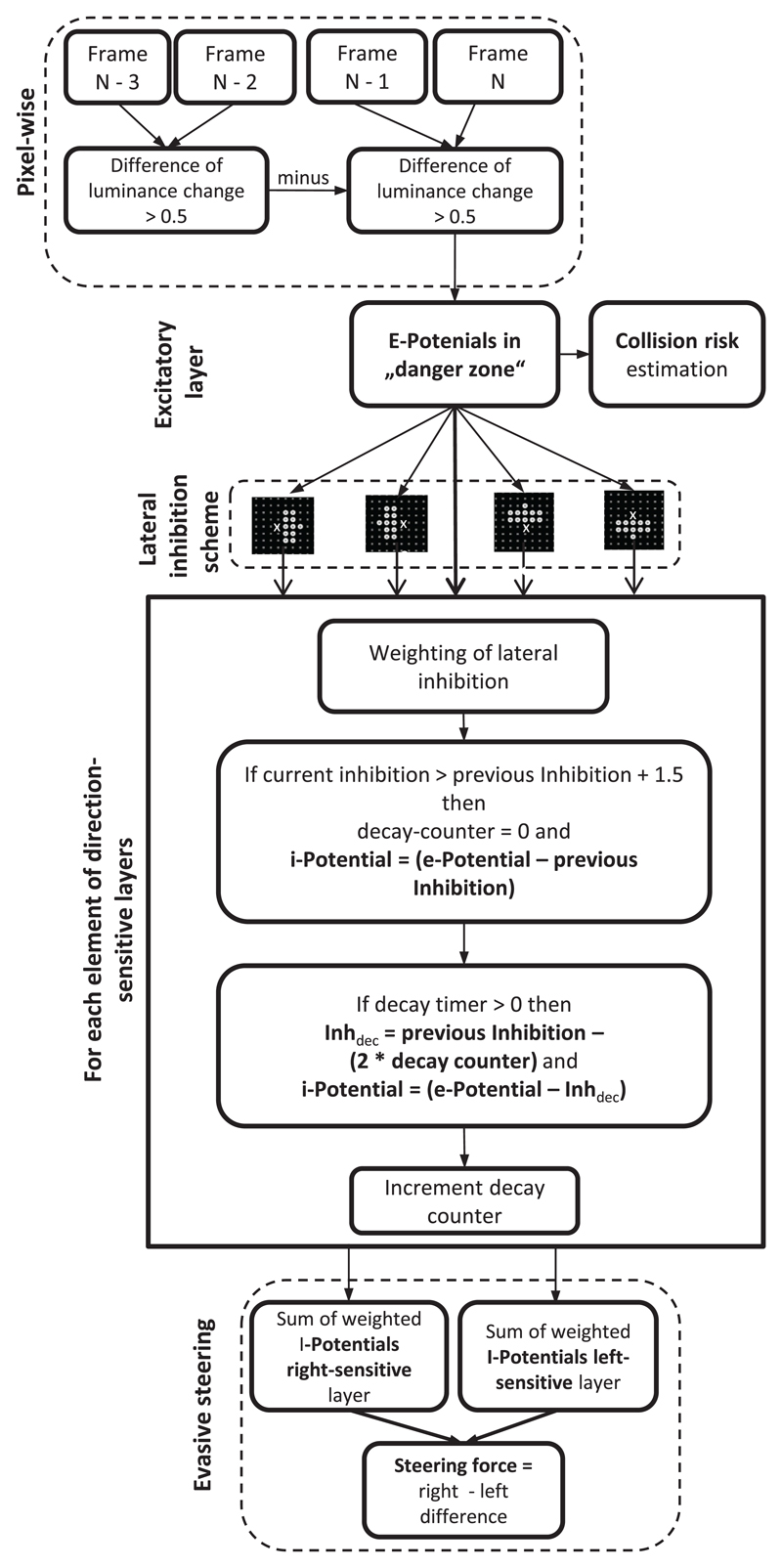
Outline of the CD algorithm and estimation of evasive steering directions; see methods for detailed description.

Figure 4 shows the details of the algorithm that was used to calculate the risk of impending collisions and evasive steering vectors. Subsequent frames of low-resolution traffic films served as input for the pixel-wise calculation of frame-by-frame luminance changes of ‘receptors’ of the ‘excitatory layer’. Each element of this layer retrieves the grey value of the local pixel of the film frame. Motion information was computed by subtracting the grey values of the current frame (*N*) from the grey values of the preceding frame (*N* − 1), whereby negative results were converted into positive ones using the ‘abs’ function. In order to increase the sensitivity of the motion detection, luminance changes that were computed from each frame pair were subtracted from the luminance change obtained from the preceding frame pair (see scheme in figure 4). The absolute difference resulting from this subtraction was stored as an ‘*e*-potential’ in the corresponding elements of the excitatory layer. The assignment of pixels to elements of the excitatory layer was 1:1. To control the influence of film contrast on the estimated collision risk, the *e*-potentials had to exceed a certain threshold to contribute to the estimation of collision risk. When films with low contrasts were used as input, this threshold was set to 0.2, whereas the threshold was 0.7 for films with higher contrasts (see table 1). The image contrast was calculated using equation (1) by taking the highest (*a*) and lowest grey values (*b*) of a representative film frame into account.

(1) $$\text{contrast}=\frac{\left(a-b\right)}{\left(a+b\right)}$$

The threshold for the *e*-potentials was calculated on the basis of the film contrast using the empirically determined equation (2) (see table 1). This threshold was left unchanged for the whole film.

(2) threshold=(contrast∗0.71)+0.378

### Calculation of collision risk

A primitive kind of object recognition was achieved by applying the rule that the number of excited elements (*e*-potentials > threshold), counted in a circle with a radius of 3 elements, had to be higher than 10 in order to maintain the *e*-potential of the focal element. If the focal element was surrounded by fewer excited elements, its *e*-potential was set to zero.

The collision risk was calculated in two steps. First, the sum of the *e*-potentials that exceeded the *e*-potential threshold was multiplied by a weighting factor that mirrored the relative excitation of the elements within the danger zone (see equation (3)).

(3) $$R_{\text{coll}}=\frac{w}{s}*\sum_{K=0}^{S}E_{k}\;|\;E_{k}>\text{Threshold}$$

Equation (3) describes the first term of the estimated collision risk, whereby *E_k_* is the *e*-potential of the *k*th element, *w* is the sum of strongly excited elements and *s* is the total number of elements belonging to the ‘danger zone’. The estimation of the impending collision risk was further improved in a second step by taking the distance of excited elements (i.e. those with *e*-potentials > 0.5) to the center of the danger zone into account. Here, I assumed that excited elements near the center of the danger zone were more critical for the estimation of impending collisions as compared to elements located near its border. Therefore, the distance factor *R*_dist_ was calculated according to equation (4) by adding the inverse values of the distance to the center of the ‘danger zone’ (*d*_k_) of all elements with *e*-potentials higher than the *e*-potential threshold.

(4) $$R_{\text{dist}}=10*\sum_{K=0}^{N}\frac{1}{d_{k}}\;|\;d_{k}>0\;\text{and}\;d_{k}<\frac{r}{2}$$

In equation (4), *N* refers to the number of excited elements with distances less than the half width of the radius *r* of the danger zone. If *N* was higher than 15, *R*_dist_ was added to *R*_coll_ to estimate the impending collision risk; otherwise, the collision risk was estimated using *R*_coll_. The factor 10 in equation (4) denotes a gain value that allowed control over the contribution of *R*_dist_ to the estimated collision risk. A gain value of 10 led to a contribution of *R*_dist_ to 37% of the maximum collision risk in the car crash (see figure 4) and to 55%, in the rear-end collision (see figure 7).

**Figure 5:**
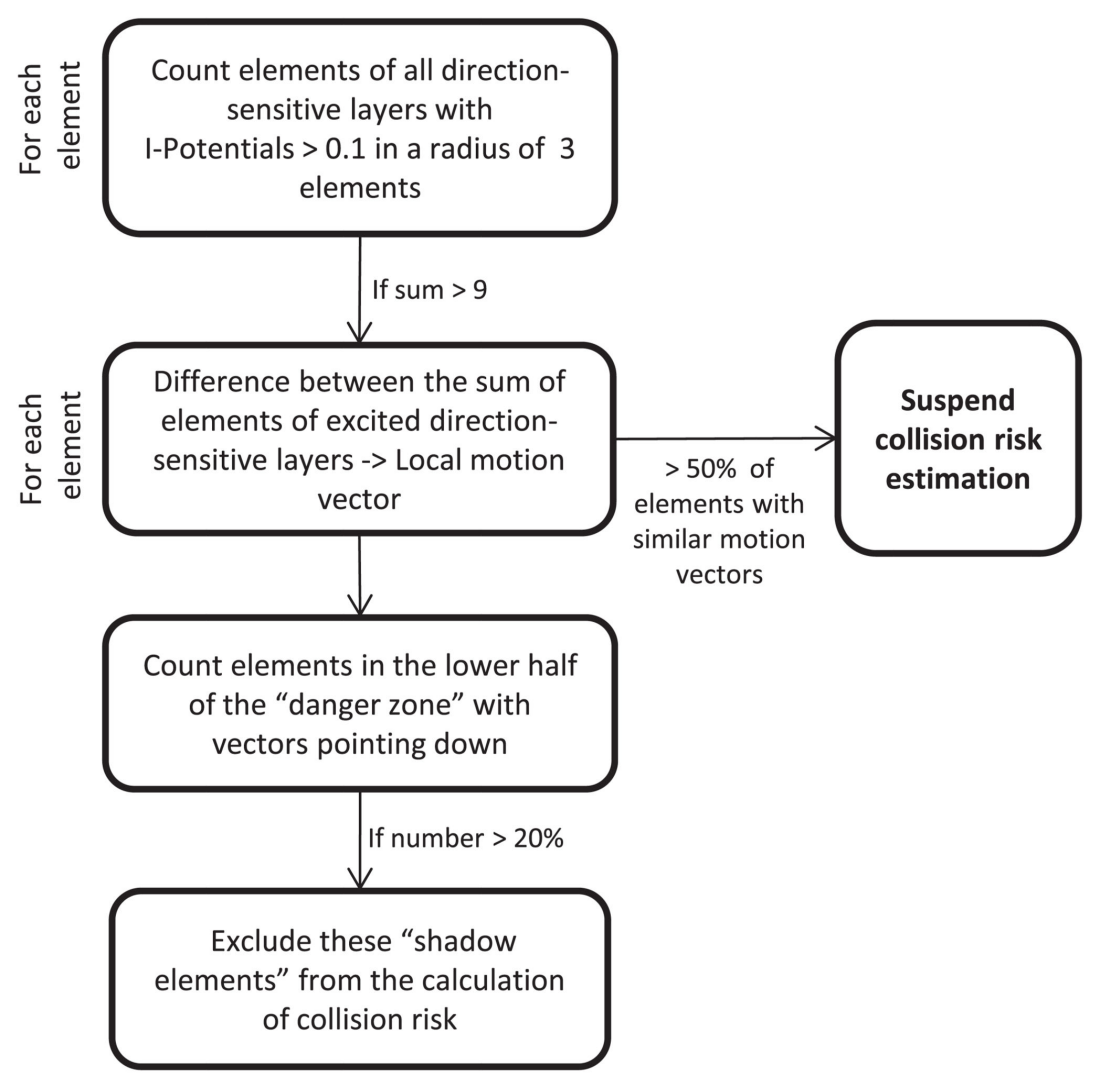
Outline of rules used to suppress shadow and motion artifacts.

In order to avoid the overstimulation in the CD algorithm, the calculation of the impending collision risk is suspended if more than 40% of all elements in the danger zone exhibited *e*-potentials that were higher than 0.5. Suspension also occurs if more than 50% of all local motion vectors (see below) indicate a similar motion direction (±45°). These rules prevent the inclusion of undesired responses to motion artifacts that arise due to self-generated movements or camera shake (see also figure 5).

### Lateral inhibition

In order to calculate local motion, the function of a lateral inhibition network was simulated according to the principle of elementary motion detectors (see figure 2; Rind and Bramwell 1996). Instead of simulating the complexity of a lateral inhibitory network by including such details as membrane potential, action potential and synaptic weight (see Yue and Rind (2009, 2013a, 2013b)), a rather simple algorithm was used (for a similar approach see Blanchard *et al* (2000); figure 4). *e*-potentials that were stored in the elements of the excitatory layer were passed on to corresponding elements of four ‘inhibitory layers’ with the same dimensions as the excitatory layer (see also Stafford *et al* (2007)). The sensitivity to motion direction of the inhibitory layers was determined by the relative position of elements that generated a delayed inhibitory input on the focal element (indicated with an ‘*X*’ in figure 4). Lateral inhibition was simulated by subtracting the sum of weighted *e*-potentials (obtained from 11 elements within a hemicircle) from the current *e*-potential of the focal element after a time delay of one processing step. When these inhibitory elements are located on the right-hand side of a focal element, this layer is sensitive to motion on the right-hand side and vice versa. If these elements are located above the focal element, this layer is sensitive to upward motion and vice versa. The *e*-potentials stored in 11 elements were first distance-weighted by dividing the *e*-potential of each element by its distance (d) from the focal element. Then, the sum of the distance-weighted *e*-potentials was multiplied by the weighting factor 0.35 according to equation(5), whereby *r_i_* is the radius of the hemicircle in which elements contribute to the inhibition (⩽3 elements).

(5) $$I_{\text{dist}}=0.35*\sum_{K=0}^{10}\frac{E_{k}}{d_{k}}\;|\;d_{k}>0\;\text{and}\;d_{k}⩽r_{i}$$

Distance-weighted inhibitory values decayed with time. The decay of inhibition of each element was calculated according to equation (6) by subtracting a value two times the decay counter from the *I*_dist_ of the preceding processing step (see figure 4).

(6) $$I_{\text{decay}}=I_{\text{dist}}-(\text{counter}*2)$$

Decayed inhibitory values were saved in the focal elements of each ‘inhibitory layer’ and subtracted from the current *e*-potential in the subsequent processing step (decay counter >0) (see equation (7)). The resulting value was stored as an ‘*i*-potential’ in each element of the ‘inhibitory layer’, whereby negative i-potentials were set to 0.

(7) $$I_{\text{potential}}=E-I_{\text{decay}}$$

If the current inhibitory value of any element is higher than the decayed inhibitory value of the last processing step plus a threshold (1.5), the ‘decay counter’ is set to 0 and the current inhibitory value is saved to become available for the processing of lateral inhibition in the next processing step.

### Calculation of evasive steering vectors

The direction and force of evasive steering vectors were estimated by calculating the difference in the excitation between the right and left motion-selective layers. The excitation of each layer was calculated using equation (8) by adding up all *i*-potentials that were higher than 0.1 and multiplying the result by a weighting factor. This weighting factor was the ratio of the number of inhibitory elements with *i*-potentials higher than 1.0 (*m*) and the number of elements belonging to the danger zone (*s*).

(8) $$E_{\text{layer}}=\frac{m}{s}*\sum_{K=0}^{S}I_{\text{potential}}|\;I_{\text{potential}}>0.1$$

The difference between the excitation of the right and left motion selective layer corresponded to the lateral object motion and had to exceed a threshold of six in order to estimate the plausible steering direction for evasive action. Stronger excitation of the right-selective layer indicated an evasive steering maneuver to the left and vice versa. The relative difference between the excitation of the right and left selective layers could be used to calculate the force of the evasive steering (see equations (9) and (10)).

(9) $$S_{\text{left}}=(E_{\text{layer}\_\text{right}}-E_{\text{layer}\_\text{left}})>6$$

(10) $$S_{\text{right}}=(E_{\text{layer}\_\text{left}}-E_{\text{layer}\_\text{right}})>6$$

### Calculation of local motion direction to suppress artifacts

Figure 5 illustrates the processing steps taken to suppress the undesired systemic responses to artifacts that originated from camera shake, self-generated movements and approaching ground shadows. The local direction of motion was estimated for each element by comparing the number of excited inhibitory elements in all four direction-selective layers within a circle with a radius of 3 elements. If this circle contained more than 9 elements with *i*-potentials that were higher than 0.1, then the local motion vector was calculated for the focal element, otherwise its calculation was suspended. The calculation of local direction of motion was based on the difference in the number of inhibitory elements in all four inhibitory layers. As soon as the number of elements in one layer exceeded the number of elements present in all other layers by four, the direction of this layer dominated. If the number of elements in two layers exceeded this threshold and their preferred directions deviated by only 90°, the local motion vector was the average between dominating inhibitory layers. The application of these simple rules allowed calculating local motion vectors with 45° precision.

If more than 50% of elements with local motion vectors indicated a similar direction (±45°), the collision detection system is suspended to prevent undesired responses of the CD algorithm to self-generated movement and camera shake. In addition, the estimation of local motion vectors enabled the suppression of motion artifacts that were caused by quickly approaching ground shadows. Elements were categorized as ‘shadow elements’ if more than 20% of all the elements in the lower half of the danger zone had a motion vector that pointed towards the lower border of the frame with ±45° precision. These elements were excluded from the CD algorithm by setting their *e*-potentials and *i*-potentials to zero.

## Results

**Figure 6:**
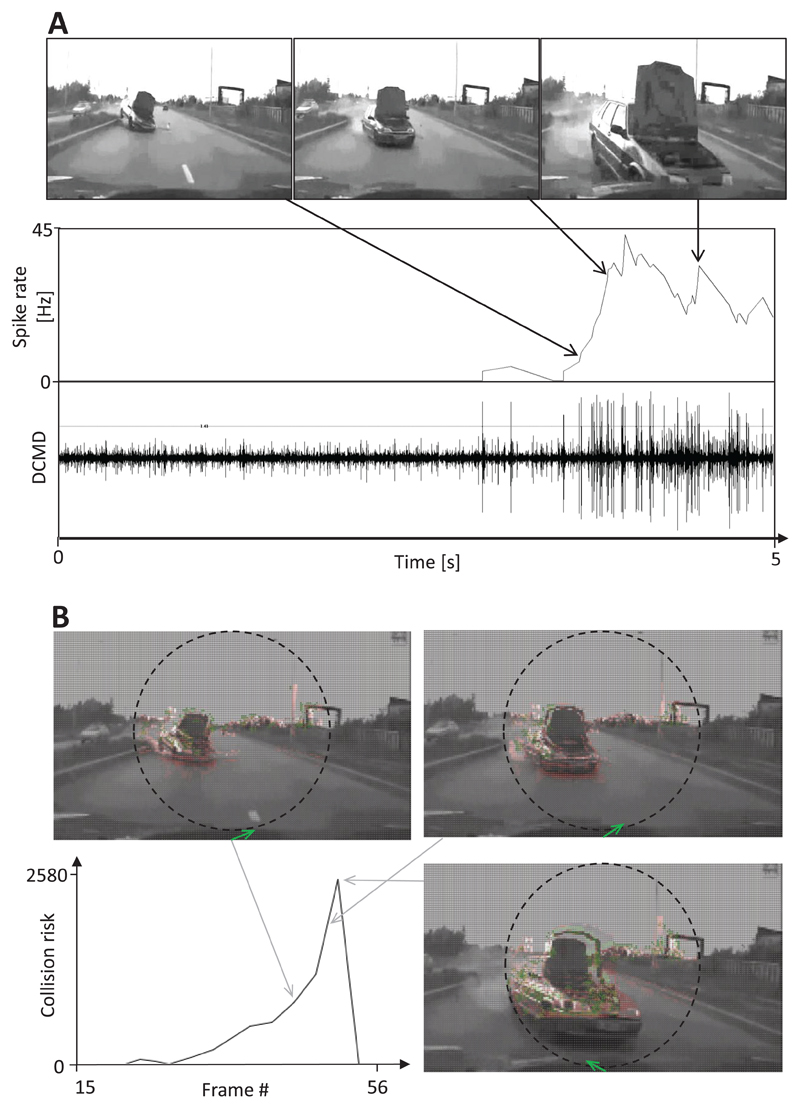
Response of the DCMD neuron and CD algorithm to an impending frontal collision. Images from video footage: a car crossed the border separating the highway lanes and crashed into the car containing the camera. (A) The DCMD neuron of a locust responded with bursts of action potentials to the impending collision (large spikes in the nerve recording in the bottom trace). The average spike frequency is shown in the panel above. Note that the DCMD response declined in the final approach phase. (B) The estimated collision risk quickly increased as the car containing the camera approached an obstacle. The estimation of collision risk was suspended during the final approach phase. Large green arrows at the bottom of images indicate a possible evasive steering direction. Red pixels indicate motion, while small green arrows represent local motion vectors.

**Figure 7:**
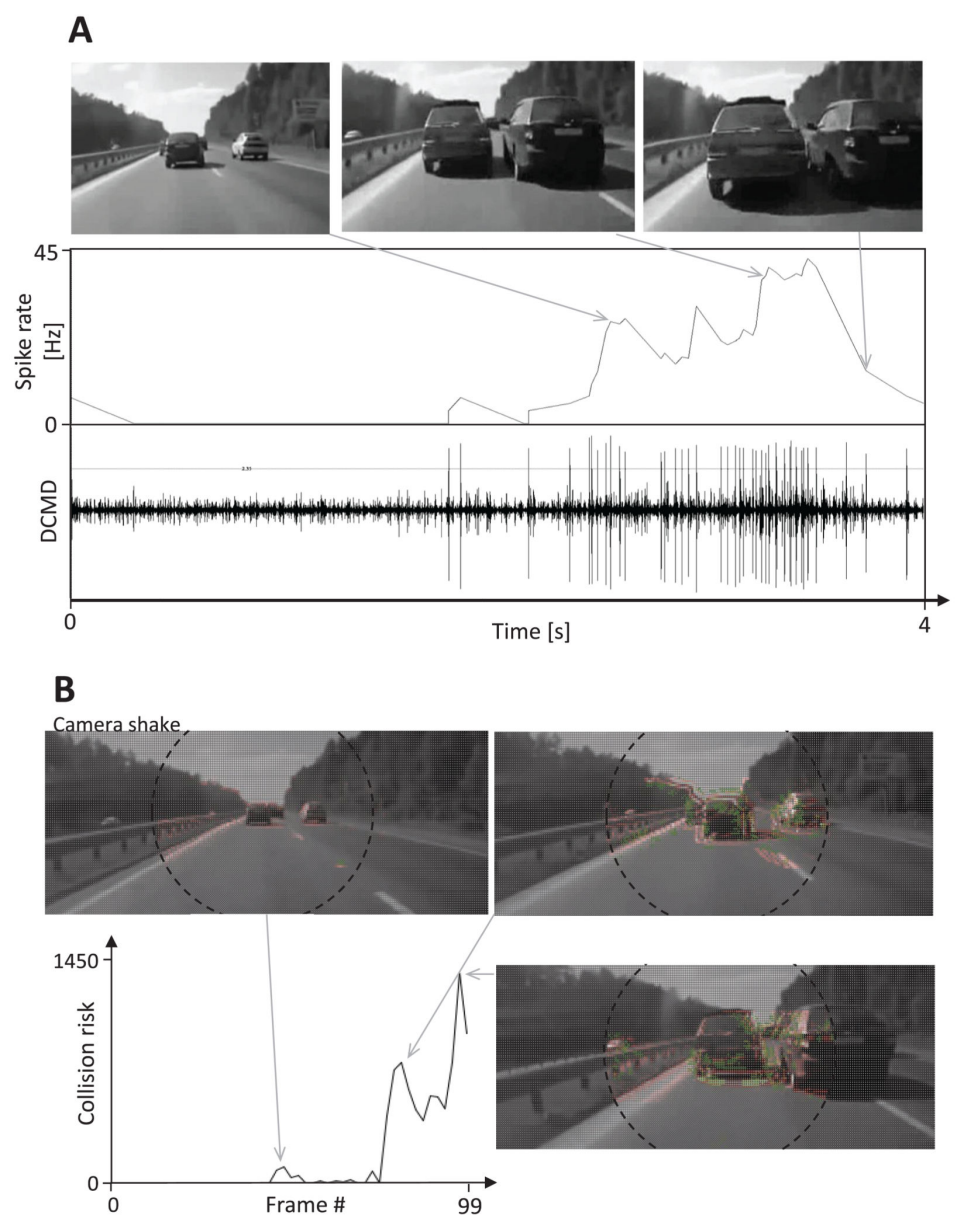
Response of the DCMD neuron and CD algorithm to an impending rear-end collision. Images from video footage: the car on the left moved to the right lane and a stationary car appeared in the left lane shortly before the car mounted with a camera crashed into it. (A) The rapid lane change of the car on the left elicited an initial action burst of action potentials and the impending collision, a second burst. In the final stage of this film, the DCMD response rapidly declined. (B) The estimated collision risk of the CD algorithm slightly increased in response to camera shake. Later, it strongly increased when the car changed lanes and a second time during the collision. The calculation of the collision risk was suspended two frames before the crash occurred. Note that it was not possible to calculate an evasive steering maneuver for this film.

The recording of the DCMD activity of locusts during the presentation of traffic films revealed that objects that seemingly approached the locust on a collision course evoked a burst of action potentials (i.e. ‘spikes’). The spiking rate strongly increased during the approach phase and dropped as soon as the object came close to the camera (see representative examples shown in figures 6(A) and 7(A)). Taking the same film as input for the CD algorithm yielded a similar result: the perceived collision risk increased as an object approached and was reduced as the frontally approaching vehicle passed close by the camera (figures 6(B) and 7(B)). The final drop of estimated collision risk is the consequence of the rule that prevents the CD algorithm from factoring in undesired responses to camera shake and self-generated motion. Since the driver of the car did not respond to the approaching car in the filmed traffic scenes shown in figures 6 and 7, a frontal collision was inevitable. In the example shown in figure 6(B), as the car broke through the highway divider, the evasive steering vector calculated was directed to the right (shown as green arrows at the bottom of images), but as the approaching car came closer, the calculated evasive steering direction changed.

**Figure 8:**
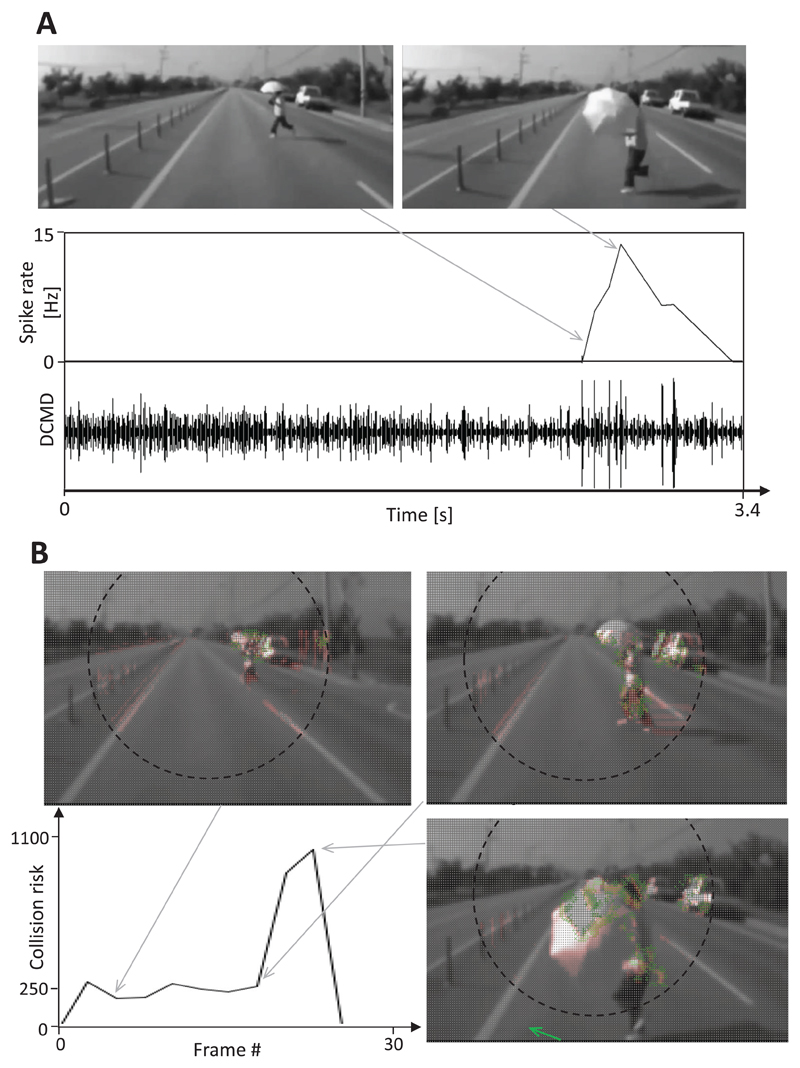
Response of the DCMD neuron and CD algorithm to an impending crash with a pedestrian. Images from video footage: a pedestrian crossing two highway lanes was hit by the car containing the camera. (A) A burst of action potentials was measured for the DCMD neuron as the car with the camera approached the pedestrian. This neuronal response was suppressed during the final phase of the film. (B) The estimated collision risk slightly increased in response to gentle camera shake. Later, it quickly increased when the pedestrian was on a collision course with the vehicle. The calculation of collision risk was suspended two frames before the crash occurred. Note that the frame rate of the original movie was only 14 Hz and 20% of the left side of the film frames is not shown.

The traffic situation shown in figure 7 is more complex since the car in the left lane needed to evade a parked car that appeared in the lane. Bursts of spikes were recorded from the DCMD neuron in response first to the lane change and second, to the rapidly approaching stationary car (see example shown in figure 7(A)). Similarly, the collision risk calculated with the help of the CD algorithm increased strongly up to a value of 740 as the car changed lanes. When the stationary car appeared in the right lane, the calculated collision risk increased to a maximum of 1320 (figure 7(B)). It was not possible to calculate an evasive steering vector for this example because the excitation levels of the right and left direction-selective layers were similar. A burst of spikes from the DCMD neurons of the locusts was also recorded when the car containing the camera crashed into a pedestrian who was crossing the two-lane highway (figure 8(A)). However, this DCMD response was rather weak (15 Hz spike rate), which may have been due to the low frame rate of the original movie (14 Hz), which leads to duplicate frames in the ‘29 Hz movie’ that was used as stimulus. The collision risk estimated using the CD algorithm increased moderately as the pedestrian entered the danger zone (figure 8(B)). Later, the collision risk increased strongly up to a maximum of 1000, and the calculated evasive steering vector indicated a quick turn to the left.

**Figure 9:**
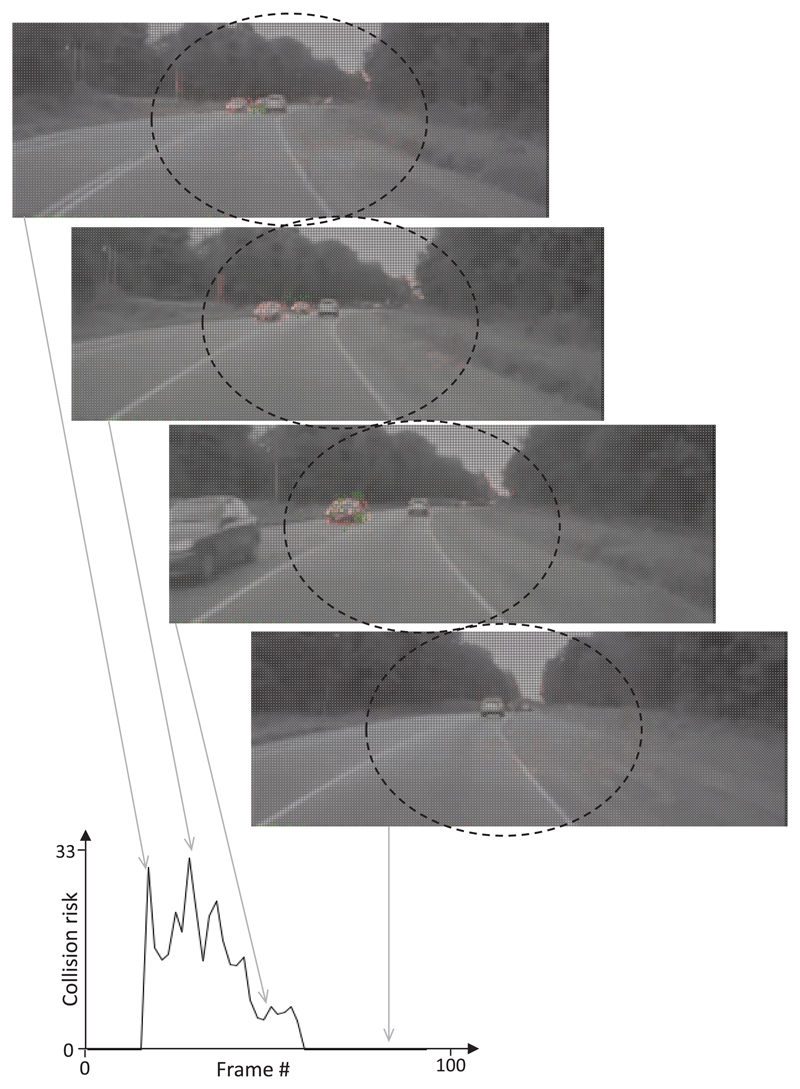
Response of the CD algorithm to on-coming traffic. Images from video footage: two cars in on-coming traffic passed the car containing the camera, which resulted in a slight increase in the estimated collision risk. In contrast, travelling behind another vehicle did not increase this risk (picture at the bottom).

**Figure 10:**
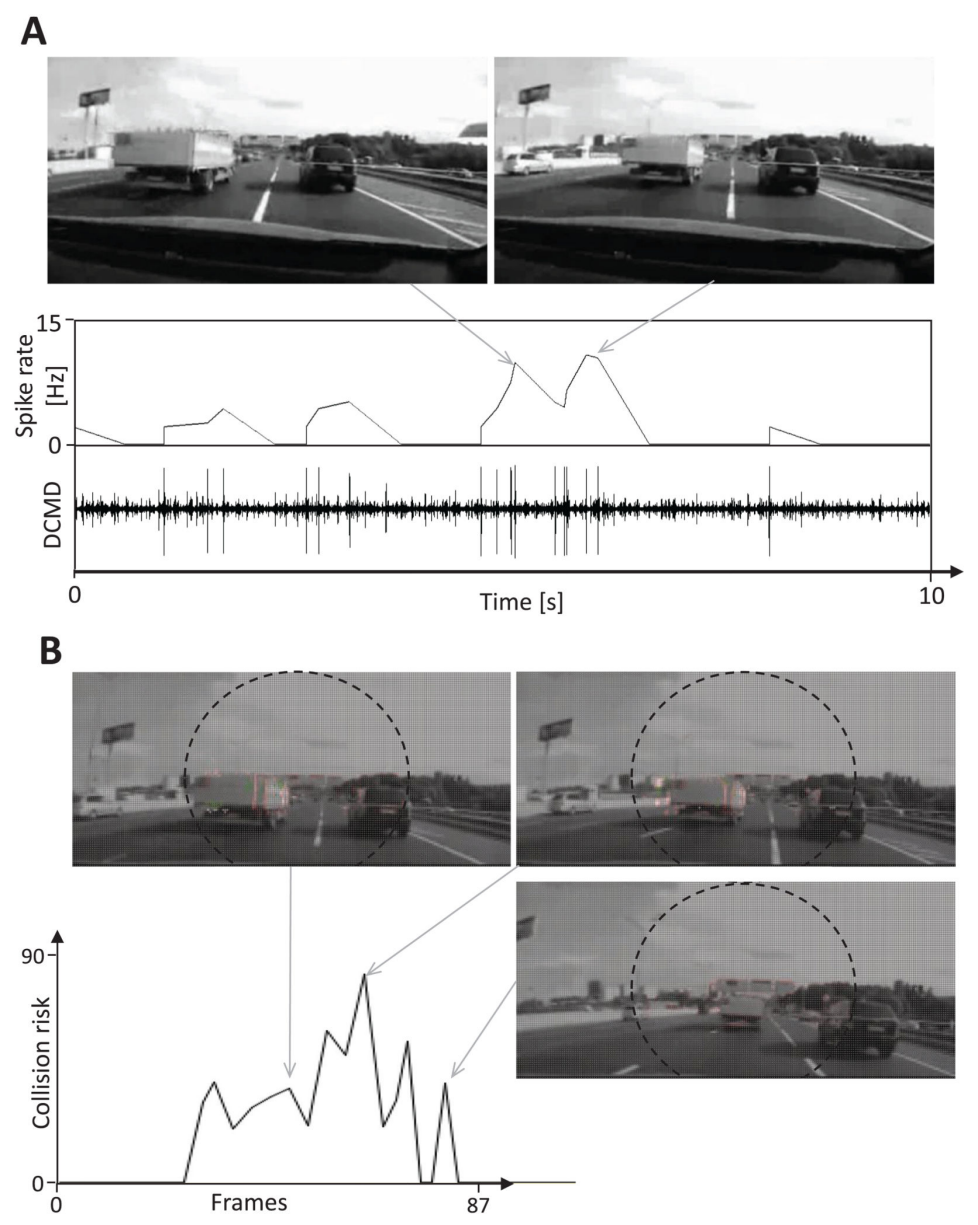
Response of the DCMD neuron and CD algorithm to a passing truck. Images from video footage: a truck overtook the car containing the camera and evoked a weak firing burst response from the DCMD neuron (A) and in the CD algorithm (B).

In contrast to these films involving crashes, the approach of on-coming traffic during a right-hand turn evoked only weak DCMD responses with spike frequencies below 5 Hz (data not shown). Similarly, the calculated collision risk of the CD algorithm remained below 30 when the approaching cars passed by safely (figure 9). Furthermore, when a truck entered the highway, the calculated collision risk of the CD algorithm slightly increased up to a maximum value of about 85 (figure 10(B)) and evoked a moderate DCMD response in locusts, resulting in a maximum spike rate of 12 Hz (see representative example in figure 10(A)). To estimate the collision risk when the car containing the camera rather aggressively passed a car that was driving slowly on the two-lane highway, a film that showed a simulation of this scene was used as input for the CD algorithm. In this virtual passing situation, the collision risk increased to a maximum of 112, and the maximum DCMD neuronal discharge was 15 Hz (average of 10 film presentations).

### False positive responses to shadows and overhead signs

**Figure 11:**
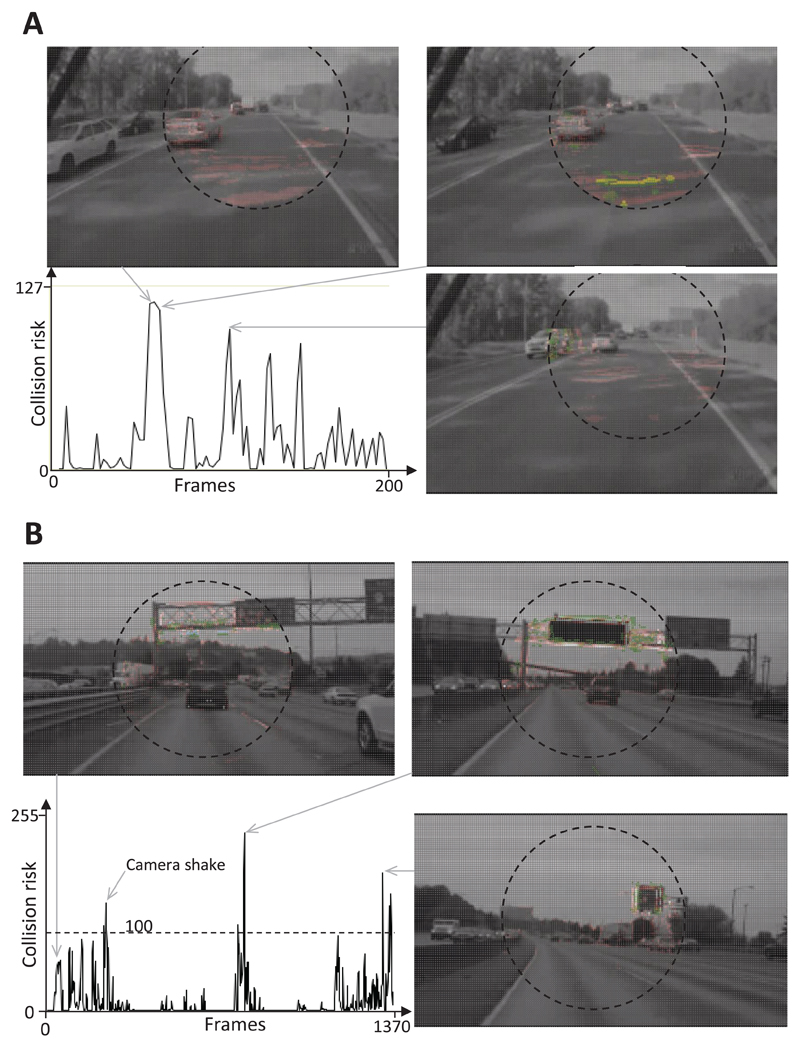
Response of the CD algorithm to ground shadows and overhead signs. (A) Collision risk slightly increased in response to a passing car and quickly approaching ground shadows. Yellow elements were identified as parts of ground shadows and were excluded from the estimation of collision risk. (B) During high-speed highway travel, the presence of camera shake and overhead signs led to a moderate increase in the estimated collision risk.

When the car drove along a street and ground shadows with high contrast levels were present, responses were frequently evoked in the CD algorithm. In order to suppress these undesired responses, elements categorized as ‘shadow elements’ were excluded from the collision risk estimation. When ground shadows were not excluded a moderate collision risk of 120 was calculated when a car passed the car containing the camera (figure 11(A)). Ground shadows were suppressed (yellow elements in figure 11(A)) in the next processing step, which prevented the algorithm from a further increase of the estimated collision risk.

During a high-speed ride on a highway (Interstate 5 in Washington) that lasted 45 s, the estimated collision risk was rather low (<100). However, as soon as overhead signs appeared in the viewing field, the estimated collision risk increased to values between 120 and 240 (figure 11(B)). In this situation, a burst of 4–10 action potentials were measured from the DCMD neurons of the locusts, which corresponds to a spike frequency maximum of 10–15 Hz.

## Discussion

Rind and Simmons (1992) observed that DCMD burst firing could be recorded in locusts that were shown scenes from a Star Wars movie that included rapidly moving objects of various sizes. In the present study, I demonstrated for the first time that DCMD of locusts shows a bursting response to objects in complex traffic scenes that were on collision trajectories. This response is remarkable because the speed of camera-equipped vehicles was much higher than the flight speed of the locusts, and the quality of the traffic movies with respect to their compression rate, image resolution and frame rate was rather low. In contrast, locusts shown film segments that included common traffic situations such as various passing maneuvers, the approach of vehicles in the oncoming lane and high-speed rides on a highway, the firing rate of DCMD remained rather low (<15 Hz). These results strongly suggest that bionic approaches can be both effectively and reliably used to develop camera-based crash avoidance detectors. Notably, the DCMD firing rate often declined during the final approach phase of an opposing object (see figures 4(A), 5(A) and 6(A)). This is most likely due to inhibition mediated by the activity of wide-field motion detection neurons (Rind and Simmons 1992) which seems to protect DCMD neurons from overstimulation and suppress neuronal responses to whole-field luminance changes.

Instead of simulating elaborate neuronal networks (Yue and Rind 2006b, 2013a, 2013b) or applying complex algorithms to discriminate objects against optical flow (Kolodko and Vlacic 2005), a simple CD algorithm was developed during this study that could be used to estimate the risk of impending collisions, despite the fact that the speed of the cars containing the cameras was high and the film image resolution and frame rate were low. The estimated collision risk strongly increased when objects rapidly approached along a collision course, whereas the collision risk estimated for everyday traffic situations was low (figures 9–11). This rapid increase in the collision risk estimated during the virtual frontal approach of an object mirrored the burst firing rate of DCMD neurons of locusts that viewed approaching objects in traffic films. The results obtained while taking this combined approach suggests that the detection of motion within a danger zone is already sufficient to simulate DCMD response and to estimate collision risk properly. The results of this study have significant implications with respect to the development of bio-inspired collision detector systems because, in a real vehicle, information about the velocity and steering direction are readily available which enables dynamic calculation of the size and position of the ‘danger zone’ within the panoramic field of view of a dashboard or windscreen camera. Theoretically, neuronal processing of optical flow and/or re-afferent feedback may restrict the field of view in flying locusts in which the response of the DCMD neurons to looming objects is suppressed while at the same time excitation in the remaining ‘physiological danger zone’ may correspond to collision risk. Indeed, there is evidence for the habituation of the DCMD response in locusts evoked by repetitive visual stimulation and dishabituation upon presenting a novel stimulus (Bacon *et al* 1995). Furthermore, a patterned background flowing outwards from the centre of the screen merely effected the LGMD’s responses to a looming stimulus as soon as the object exceeded about 24° (Gabbiani *et al* 2002), which suggests the existence of something analogue to a danger zone in the optic lobe of locusts.

One potential drawback of the ‘danger zone’ is its low degree of sensitivity to objects that rapidly approach from the side of the vehicle because it is not evaluating the direction and speed of objects in the surrounding traffic scene. Such situations can occur, for example, at road crossings, intersections and city traffic. Nevertheless, this algorithm can be used to detect impending collisions when vehicles are driven at high speeds on highways and motorways. Therefore, this CD algorithm may extend the functionality of commercially-affordable ‘screen cameras’ that detect objects on a collision course at low driving speeds (i.e. vision-based crash avoidance systems of modern cars). However, due to the low level of image resolution of films that were used in this study, the response to small objects such as children or animals will be delayed. A higher image resolution will help to overcome this problem, but makes it necessary to tune some parameters of the CD algorithm to achieve a similar sensitivity to objects on a collision course and to estimate the direction of motion. Using high resolution frames makes is necessary to adjust the size of the circle that is used for a primitive kind of object recognition before estimating the collision risk (currently a radius of 3 elements). Additionally, it is necessary to adjust the hemicircle for the calculation of the delayed inhibitory input and the weighting factor in equation (5). When films of high resolution are used as input, the processing speed of the CD algorithm may be improved by using a superpixel method (for a practical application of this method see Morerio *et al* (2014)).

Another weakness of this CD algorithm is its possible response to self-generated motion and camera shake. The first aspect, however, can be suppressed by estimating local motion vectors (see figure 3) and the latter aspect, by using an image stabilizer that compensates shakes of the CD camera. False positive responses to overhead signs and ground shadows (figure 11(B)) indicate that it would be advisable to combine this CD method based on visual cues with a proximity sensor (e.g. a radar system). Kim *et al* (2005) and Spagnolo *et al* (2006) described algorithms that classify the foreground from the background in films of stationary cameras. A modification of these algorithms may help to suppress undesired responses of the CD method to objects belonging to the background.

Surprisingly, differential excitation levels that were measured between the right and left direction-selective layers allowed the estimation of a plausible steering direction that could be used during an evasive maneuver and help prevent the car from crashing into an approaching object (green arrows in figure 6). In the example of the crash with a pedestrian shown in figure 8, the estimated collision risk increased after a brief period of delay, and an evasive steering direction was calculated once it was already too late for an evasive steering maneuver. These results suggest that a frame rate of only 14 Hz is too low to ensure the reliable performance of this CD algorithm.

The estimated collision risk can be used as input for a brake assistant and converted into braking force. Furthermore, the estimated evasive steering direction can be used as input to control the car steering in a dangerous situation. Therefore, this insect-inspired CD algorithm would likely have prevented the crash that was observed at the end of traffic films shown in figures 6 and 7. This, however, raises the question of the threshold that must be exceeded prior to the activation of a driver-assistant system. This threshold must be higher than the collision risk estimated during the approach of ground shadows, camera shake and on-coming traffic to discriminate between dangerous and harmless traffic situations. In the traffic examples shown in figures 6–11, a driver-assistant system would have needed to take control of the car when the collision risk estimate exceeded a value of about 150–200.

## Conclusions

The definition of a ‘danger zone’, in which collisions on a highway or motorway are most likely, enables the application of a rather simple motion detection algorithm to estimate the risk of impending collisions, which is encoded in the activity of a collision-detector neuron in locusts. This CD algorithm is based on pixel-wise changes in luminance and takes the distance between ‘excited pixels’ and the center of the ‘danger zone’ into account. By combining both measures, it was possible to estimate the risk of collisions in various traffic situations such as impending frontal collisions and a rear-end collision. In contrast, the collision risk estimated during passing maneuvers, high-speed highway travel and the approach of on-coming traffic in the opposing lane was rather low.

The simple simulation of the function of lateral inhibition networks, which are sensitive to four different directions of motion, allowed the calculation of local motion vectors. Evaluation of these vectors in a small image region surrounding each pixel was sufficient to suppress artifacts due to the presence of shadows on the ground and responses to self-generated motion. Furthermore, the differential excitation levels measured between the right and left direction-selective layers could be used to estimate evasive steering vectors. In the future, this CD algorithm can be applied to estimate collision risk and calculate evasive steering vectors to control a driver-assistant system that translates the estimated collision risk into braking force, and the evasive steering vectors into steering force and direction. However, in order to prevent undesired responses to ground shadows and overhead signs, the CD camera should be combined with a proximity sensor based on radar waves.

Much of this CD algorithm is executed in a pixel-wise manner or including elements that mimic the function of neurons in a simple lateral inhibitory network. Therefore, it is possible to realize the main parts of this algorithm in hardware solutions (e.g. VLSI technology and FPGA chips) that allow parallel processing of collision risk and evasive steering even when CD cameras are operating at high frame rates (e.g. Aubépart and Franceschini (2007)).

## Figures and Tables

**Table 1:** Average brightness of films measured at the position of the locust, image resolution, dimension of ‘danger zone’ and the threshold of grey values used for the estimation of collision risk.

Movie name	Brightness (lux)	Figure number	Grey value threshold	Image contrast	Radius ‘danger zone’ (pixel)	Image resolution netlogo (pixel)
Crash on the highway	69	4B	0.4	0.66	50	200 × 110
Rear-end collision	54	5B	0.7	0.85	50	204 × 80
Pedestrian crash	51	6B	0.5	0.76	50	204 × 94
On-coming traffic	58	7	0.2	0.49	50	200 × 72
Truck passing on a highway	66	8B	0.7	0.87	45	204 × 90
Passing with on-coming traffic	59	9A	0.7	0.87	50	200 × 122
High-speed highway travel	69	9B	0.5	0.76	45	200 × 114
Simulated passing	79	not shown	0.4	0.68	50	200 × 82
